# In Silico Prediction of Drug–Drug Interactions Mediated by Cytochrome P450 Isoforms

**DOI:** 10.3390/pharmaceutics13040538

**Published:** 2021-04-13

**Authors:** Alexander V. Dmitriev, Anastassia V. Rudik, Dmitry A. Karasev, Pavel V. Pogodin, Alexey A. Lagunin, Dmitry A. Filimonov, Vladimir V. Poroikov

**Affiliations:** Laboratory of Structure-Function Based Drug Design, Department of Bioinformatics, Institute of Biomedical Chemistry, Pogodinskaya Str. 10, bldg. 8, 119121 Moscow, Russia; rudik_anastassia@mail.ru (A.V.R.); dmitry.karasev@ibmc.msk.ru (D.A.K.); pogodinpv@gmail.com (P.V.P.); alexey.lagunin@ibmc.msk.ru (A.A.L.); dmitry.filimonov@ibmc.msk.ru (D.A.F.); vladimir.poroikov@ibmc.msk.ru (V.V.P.)

**Keywords:** drug interaction, DDI, computational prediction, in silico, QSAR, drug metabolism, ADME, pharmacokinetics, CYP, polypharmacy, metabolic DDI, P450, 1A2, 2B6, 2C19, 2C8, 2C9, 2D6, 3A4

## Abstract

Drug–drug interactions (DDIs) can cause drug toxicities, reduced pharmacological effects, and adverse drug reactions. Studies aiming to determine the possible DDIs for an investigational drug are part of the drug discovery and development process and include an assessment of the DDIs potential mediated by inhibition or induction of the most important drug-metabolizing cytochrome P450 isoforms. Our study was dedicated to creating a computer model for prediction of the DDIs mediated by the seven most important P450 cytochromes: CYP1A2, CYP2B6, CYP2C19, CYP2C8, CYP2C9, CYP2D6, and CYP3A4. For the creation of structure–activity relationship (SAR) models that predict metabolism-mediated DDIs for pairs of molecules, we applied the Prediction of Activity Spectra for Substances (PASS) software and Pairs of Substances Multilevel Neighborhoods of Atoms (PoSMNA) descriptors calculated based on structural formulas. About 2500 records on DDIs mediated by these cytochromes were used as a training set. Prediction can be carried out both for known drugs and for new, not-yet-synthesized substances. The average accuracy of the prediction of DDIs mediated by various isoforms of cytochrome P450 estimated by leave-one-out cross-validation (LOO CV) procedures was about 0.92. The SAR models created are publicly available as a web resource and provide predictions of DDIs mediated by the most important cytochromes P450.

## 1. Introduction

For the treatment of complex disorders, patients often take multiple medications at the same time, which potentially cause drug–drug interactions (DDIs). Usually, DDIs are divided into three types: pharmaceutical, pharmacodynamic, and pharmacokinetic [1]. Pharmaceutical DDIs may appear due to physical or chemical interactions, for example, when drugs are mixed in a syringe before infusion, and such DDIs are rare. Pharmacodynamic DDIs may occur when a pair or more co-administered drugs act on the same physiological system or target. Pharmacokinetic DDIs are very common and occur when one of the drugs (“violator” or “precipitant” drug) affects the absorption, distribution, metabolism, or excretion of another drug (“victim” or “object” drug). Such DDIs provoke an increase or a decrease in the exposure of an object drug and lead to a change in drug pharmacological action. In this study, we focused on the pharmacokinetic DDIs at the metabolism level (biotransformation), the so-called “metabolic DDIs.”

The most common drug-metabolizing enzymes (DMEs) in the first phase of xenobiotic metabolism in the human body are several isoforms of the cytochrome P450 superfamily. The U.S. Department of Health and Human Services Food and Drug Administration Center for Drug Evaluation and Research (FDA CDER) requires determining which drug-metabolizing enzymes (CYP3A, CYP2D6, CYP2C19, CYP2C9, CYP2C8, CYP2B6, or CYP1A2) metabolize the investigational drug during in vitro studies of metabolic DDIs estimates [2].

In silico methods can help prioritize drug discovery efforts by guiding, but not replacing, in vitro and in vivo experiments. Previously, we presented a comprehensive review of the methods for predicting the DDIs related to the inhibition or induction of DMEs [3]. Most of such in silico methods predict DDIs indirectly. A recently presented machine learning (ML) method used different molecular fingerprints to classify compounds as inhibitors or noninhibitors of five major cytochrome P450 isoenzymes [4]. Ligand-based and structure-based methods dealing with substrates, inhibitors [5,6], and inducers [7] of particular DMEs. Results of prediction could help to determine possible DDIs. However, such conclusions are not sufficiently reliable, as the pairs of substances that are substrates and inhibitors (or inducers) of DMEs may not exhibit DDIs. On the other hand, DDIs have often occurred between substances that could act as substrates, inducers, and inhibitors (that may act by various inhibition mechanisms); for example, this is a widespread case for cytochrome P450 CYP3A4 [8]. At best, a pair of potentially exhibiting DDI substances should be considered together during prediction as the whole entity. However, previously developed ligand-based and structure-based computational methods did not consider two substances in pairs simultaneously. Direct DDIs estimation methods for the pairs of substances include structure resemblance and functional similarities methods and literature-based DDIs prediction methods [9,10,11,12,13,14]. These methods deal with the pairs of substances but require information about the pharmacokinetics and pharmacodynamics [9,14], interaction profile, target and side-effects [10,13], and the phenotypic, therapeutic, chemical, and genomic properties [11] of substances or medical records [12]. It is clear that for new, not-yet-synthesized, and virtual substances, such information does not exist. The results of predictions of this group of methods [9,10,11,12,13,14] have often been presented as data sets containing a bulk conglomerate of information about potential DDIs predicted between the existing drugs. Such examples include 430,128 [10], 145,068 [13], and over 250,000 [14] records of unknown potential DDIs in the sets of predicted results. However, this bulk of information concerning drug pairs is provided without assessment of the possibility of DDIs manifestation.

The current study aimed to create the computational structure–activity relationship (SAR) models to predict metabolic DDIs mediated by CYP1A2, CYP2B6, CYP2C19, CYP2C8, CYP2C9, CYP2D6, or CYP3A4. We have previously developed models for DDIs severity prediction [15,16] that used the PASS (Prediction of Activity Spectra for Substances) program and PoSMNA (Pairs of Substances Multilevel Neighborhoods of Atoms) substructural descriptors. These models were able to predict the classes of DDIs severity for pairs of molecules according to OpeRational ClassificAtion (ORCA). In the current study, we used the same methods and descriptors but implemented them to predict whether two molecules would manifest metabolic DDIs mediated by the seven cytochromes mentioned above. Due to the limited possibilities of creating an appropriate training set, the stereochemical features of molecules were not taken into account by our descriptors. In addition, in the current realization of the method, DDI predictions were obtained in qualitative mode (“YES” or “NO”). Unlike other ligand-based and structure-based methods [4,5,6,7,8], our approach operated with two substances in pairs at once. This is reasonable for the DDI phenomenon, in which two substances interact simultaneously. It gives a direct indication of DDIs for the pairs of molecules without suggestions of the role of particular compounds, which is not always obvious (without consideration of inhibition or induction of a particular enzyme). In contrast to structure resemblance, functional similarities, and literature-based DDIs prediction methods [9,10,11,12,13,14], our prediction method uses only structural formulas of compounds; it does not require any information about their biological activity. This means that our method can be applied for not-yet-investigated, new, and virtual substances. Moreover, our method provides a probabilistic assessment of possible DDIs and evaluates the possibility of DDIs manifestation for predicted pairs.

## 2. Materials and Methods

### 2.1. Information on DDIs and Training Set Creation

We used DDIs data collected from two sources of information. The first source was DrugBank Version 4.1 (University of Alberta and The Metabolomics Innovation Centre, Edmonton, AB, Canada) [17] that contains information about interactions derived from public drug databases. The second source of DDIs data was the Fujitsu ADME Database (Chemistry & Life Science Group, Fujitsu, Tokyo, Japan) [18].

The final data set includes information from both sources. It was used to create the training set containing information about 2345 pairs of single-component organic compounds that interacted due to CYP1A2, CYP2B6, CYP2C19, CYP2C8, CYP2C9, CYP2D6, or CYP3A4. The detailed information is presented in Table 1.

It is well known that CYP3A4 is the major isoform of human cytochrome P450 involved in drug metabolism and pharmacokinetic DDIs. As we can see from Table 1, the number of DDIs associated with CYP3A4 in the training set is twice as high as the number of pairs for the remaining six cytochromes. It fully reflects the real situation and illustrates that the training set is representative.

### 2.2. PASS

The PASS software (Laboratory of Structure-Function Based Drug Design, Institute of Biomedical Chemistry, Moscow, Russia) [19] is based on the advanced naïve Bayes classifier and predicts the profiles of biological activity for drug-like compounds. The PASS algorithm creates a classification model of structure–activity relationships based on the training set with structures and known biological activities of known pharmaceutical agents. The PASS prediction results are presented as a ranked list of various biological activities with calculated probabilities *P_a_* (“to be active”) and *P_i_* (“to be inactive”). The most probable activities are those predicted with the maximum value Δ*P* = *P_a_* − *P_i_*. Currently, PASS predicts more than 8000 types of biological activities, including pharmacological effects, mechanisms of action, influences on gene expression, toxic and adverse effects, and interactions with metabolic enzymes and transporters. Biological activities for particular molecules in the PASS program are represented qualitatively as “active” or “inactive.” The structural formulae of drug-like organic compounds are described by Multilevel Neighborhoods of Atoms (MNA) descriptors.

The prediction of DDIs occurring due to interactions with various cytochrome P450 isoforms is similar to the prediction of biological activity using the PASS software. For DDIs prediction mediated by cytochrome P450 isoforms, the input data are represented by the pairs of structural formulas of studied drug-like compounds. The prediction results for each pair of compounds are presented by the probabilities *P_a_* and *P_i_* lists, which estimate DDIs that may occur due to interactions with CYP1A2, CYP2B6, CYP2C19, CYP2C8, CYP2C9, CYP2D6, and CYP3A4.

### 2.3. Pairs of Substances Multilevel Neighborhoods of Atoms Descriptors

**Figure 1:**
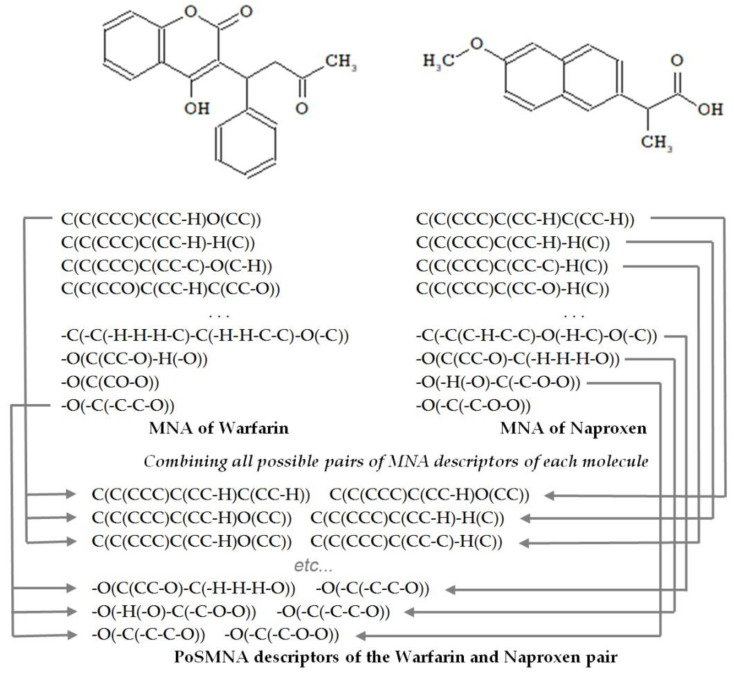
Representation of the warfarin and naproxen molecules by Pairs of Substances Multilevel Neighborhoods of Atoms (PoSMNA) descriptors.

To describe the structures of drug pairs, we used PoSMNA descriptors instead of the MNA descriptors applied in the standard PASS software version [19]. PoSMNA descriptors can be used to predict various phenomena, e.g., synergistic effects of two drugs or the prediction of DDIs. Initially, we developed and used PoSMNA descriptors to predict DDIs severity [15,16]. The set of PoSMNA descriptors is the direct product of a combination of two sets of MNA descriptors for each molecule in the DDI pair as {a,b,c,…} × {d,e,f,…} = {ad,ae,af,…, bd,be,bf,…, cd,ce,cf,…}. MNA/2 (second level of MNA descriptors) for non-hydrogen heavy atoms is used for PoSMNA creation. The MNA descriptors are ordered lexicographically for each pair of compounds, for example, from string “C(C(CCC)C(CC-H)C(CC-H)) C(C(CCC)C(CC-H)O(CC))” to “-O(-C(-C-C-O)) -O(-C(-C-O-O))” (see the examples of PoSMNA descriptors for warfarin and naproxen in Figure 1).

To create the models for DDIs prediction, PoSMNA descriptors were generated for all pairs of compounds with known DDIs mediated by CYP1A2, CYP2B6, CYP2C19, CYP2C8, CYP2C9, CYP2D6, or CYP3A4 isoforms of cytochrome P450 in the training set.

## 3. Results

To evaluate the DDIs prediction accuracy, the IAP (Invariant Accuracy of Prediction) values were calculated using leave-one-out cross-validation procedures (LOO CV). The IAP criterion is numerically equivalent to the AUC ROC (Area Under Curve of the Receiver Operating Characteristic) [19]. The IAP value is a sample estimate of the probability randomly selected from an independent test set that will correctly classify positive and negative examples. The accuracy of the prediction of DDIs caused by different isoforms of cytochrome P450 is presented in Table 2.

The developed models showed good accuracy varying from 0.82 (for CYP2C19 DDIs) to 0.98 (for CYP2C8 DDIs) with an average IAP of about 0.92. It is essential that the accuracy for DDIs mediated by CYP3A4 is high (0.93) because interactions on the level CYP3A4 can cause severe DDIs that must be detected and avoided during the investigation of new drugs. Thus, the accuracy of SAR models is adequate to use this method for practical tasks of drug discovery and development.

**Figure 2:**
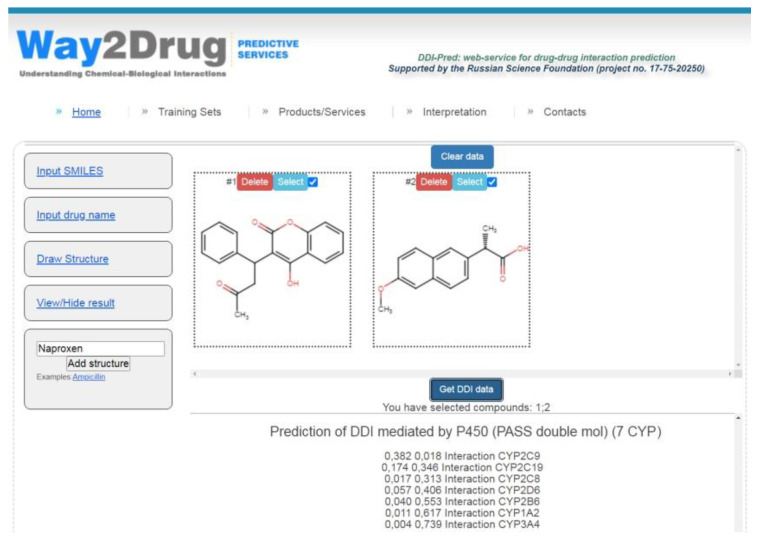
DDI prediction for warfarin and naproxen performed using the web service [20].

The models created are freely available via the Internet on the Way2Drug.com web portal on the DDIs web-service [20] that allows for the prediction of various DDIs parameters and does not require registration or log-in. The combinations of warfarin taken regularly (widely used anticoagulant with narrow therapeutic index) with various nonsteroidal anti-inflammatory drugs (NSAIDs) are common and can increase the risk of gastrointestinal bleeding [21]. As an example for illustrating the web-service analysis, the potential DDI for a pair of warfarin and naproxen (one of the commonly used NSAIDs) was predicted (see Figure 2).

The results of the prediction displayed in the block “Prediction of DDIs mediated by P450 (PASS double mol) (7 CYP)” show that the maximum Δ*P* value (0.364) was calculated for cytochrome P450 CYP2C9 (see Table 3). Therefore, the DDI for warfarin and naproxen is most likely to occur at the level of biotransformation carried out by cytochrome P450 CYP2C9.

Negative Δ*P* values for the other six isoforms of cytochrome P450 indicate that these enzymes are not involved in DDIs at the level of warfarin and naproxen biotransformation.

## 4. Discussion

Because of polypharmacy, when several drugs are taken simultaneously, the phenomenon of metabolic DDIs may appear. DDIs manifest in the mutual influence of drugs on their biotransformation, its slowdown, or acceleration, and leads to a change in the pharmacological action of drugs.

To avoid drug withdrawal from the market due to DDIs, pharmaceutical companies perform in vitro and in vivo studies. Physiologically based pharmacokinetic (PBPK) modeling is the in silico method of DDIs prediction that has already proved its applicability in the drug discovery and development process. It is clear that in silico methods will be used more intensively to reduce investigation costs [3].

The main problem we consider is the study and use of the relationship of chemical compound structure and the phenomenon of metabolic DDIs mediated by the seven isoforms of cytochrome P450 most involved in drug metabolism. The models created can be applied for virtual and not-yet-synthesized molecules using only their structural formulas. The implementation of PoSMNA descriptors and the PASS program algorithm for DDIs prediction at the level of cytochromes P450 makes it possible to consider a pair of molecules interacting as one entity without specifying the roles (substrate, inhibitor or inducer, “object” or “precipitant” drug) of particular substances in the DDI process. Such an approach is unique and has already been used to create models for DDIs severity prediction [15,16]. However, when predicting the DDIs severity without taking into account concrete pharmacokinetic or pharmacodynamic DDIs mechanisms, the accuracy of the prediction was not high enough, as compared to that obtained in the current study that considers only pharmacokinetic DDIs mediated by the seven cytochrome P450 isoforms (0.84 for three classes and 0.75 for five classes of severity vs. 0.92 for DDIs prediction mediated by cytochrome P450 isoforms). Such a lower accuracy may be explained by the unclear separation of DDIs of these severity classes among themselves and the cases of DDIs in neighboring classes in the training set and by neglecting the DDIs mechanisms. In this study, the average accuracy of DDIs prediction at the level of cytochrome P450 isoforms is higher (0.92) due to the structural specificity of substances from the pairs that interact at a particular level of the cytochrome P450 isoform. Further research should combine the prediction of DDIs severity at the level of a particular metabolic enzyme. To achieve this goal, it is necessary to expand, improve, and refine the training sets.

## Figures and Tables

**Table 1:** The number of drug–drug interactions (DDIs) mediated by various isoforms of cytochrome P450 in the training set.

Isoforms of Cytochrome P450	Number of DDIs
CYP1A2	132
CYP2B6	27
CYP2C19	80
CYP2C8	55
CYP2C9	204
CYP2D6	231
CYP3A4	1616

**Table 2:** Accuracy of the DDIs prediction.

Isoforms of Cytochrome P450	IAP
Interaction CYP1A2	0.95
Interaction CYP2B6	0.91
Interaction CYP2C19	0.82
Interaction CYP2C8	0.98
Interaction CYP2C9	0.95
Interaction CYP2D6	0.90
Interaction CYP3A4	0.93
**Average**	**0.92**

**Table 3:** DDI prediction for warfarin and naproxen at the level of cytochrome P450 isoforms.

Isoforms of Cytochrome P450	*P_a_*	*P_i_*	Δ*P*
Interaction CYP2C9	0.382	0.018	0.364
Interaction CYP2C19	0.174	0.346	−0.172
Interaction CYP2C8	0.017	0.313	−0.296
Interaction CYP2D6	0.057	0.406	−0.349
Interaction CYP2B6	0.04	0.553	−0.513
Interaction CYP1A2	0.011	0.617	−0.606
Interaction CYP3A4	0.004	0.739	−0.735

## Data Availability

Data are contained within the article.

## References

[1] Kennedy C., Brewer L., Williams D. (2016). Drug Interactions. Medicine.

[2] In Vitro Drug Interaction Studies—Cytochrome P450 Enzyme- and Transporter-Mediated Drug Interactions Guidance for Industry. https://www.fda.gov/media/134582/download.

[3] Dmitriev A.V., Lagunin A.A., Karasev D.A., Rudik A.V., Pogodin P.V., Filimonov D.A., Poroikov V.V. (2019). Prediction of Drug-Drug Interactions Related to Inhibition or Induction of Drug-Metabolizing Enzymes. Curr. Top. Med. Chem..

[4] Banerjee P., Dunkel M., Kemmler E., Preissner R. (2020). SuperCYPsPred—A web server for the prediction of cytochrome activity. Nucleic Acids Res..

[5] Hochleitner J., Akram M., Ueberall M., Davis R.A., Waltenberger B., Stuppner H., Sturm S., Ueberall F., Gostner J.M., Schuster D. (2017). A Combinatorial Approach for the Discovery of Cytochrome P450 2D6 Inhibitors from Nature. Sci. Rep..

[6] Kaserer T., Höferl M., Müller K., Elmer S., Ganzera M., Jäger W., Schuster D. (2015). In Silico Predictions of Drug—Drug Interactions Caused by CYP1A2, 2C9 and 3A4 Inhibition—A Comparative Study of Virtual Screening Performance. Mol. Inform..

[7] Torimoto-Katori N., Huang R., Kato H., Ohashi R., Xia M. (2017). In Silico Prediction of hPXR Activators Using Structure-Based Pharmacophore Modeling. J. Pharm. Sci..

[8] Fahmi O.A., Maurer T.S., Kish M., Cardenas E., Boldt S., Nettleton D. (2008). A combined model for predicting CYP3A4 clinical net drug-drug interaction based on CYP3A4 inhibition, inactivation, and induction determined in vitro. Drug. Metab. Dispos..

[9] Takeda T., Hao M., Cheng T., Bryant S.H., Wang Y. (2017). Predicting drug-drug interactions through drug structural similarities and interaction networks incorporating pharmacokinetics and pharmacodynamics knowledge. J. Cheminform..

[10] Vilar S., Uriarte E., Santana L., Lorberbaum T., Hripcsak G., Friedman C., Tatonetti N.P. (2014). Similarity-based modeling in large-scale prediction of drug-drug interactions. Nat. Protoc..

[11] Cheng F., Zhao Z. (2014). Machine learning-based prediction of drug-drug interactions by integrating drug phenotypic, therapeutic, chemical, and genomic properties. J. Am. Med. Inf. Assoc..

[12] Duke J.D., Han X., Wang Z., Subhadarshini A., Karnik S.D., Li X., Hall S.D., Jin Y., Callaghan J.T., Overhage M.J. (2012). Literature based drug interaction prediction with clinical assessment using electronic medical records: Novel myopathy associated drug interactions. PLoS Comput. Biol..

[13] Zhang P., Wang F., Hu J., Sorrentino R. (2015). Label Propagation Prediction of Drug-Drug Interactions Based on Clinical Side Effects. Sci. Rep..

[14] Ferdousi R., Safdari R., Omidi Y. (2017). Computational prediction of drug-drug interactions based on drugs functional similarities. J. Biomed. Inf..

[15] Dmitriev A.V., Filimonov D.A., Rudik A.V., Pogodin P.V., Karasev D.A., Lagunin A.A., Poroikov V.V. (2019). Drug-drug interaction prediction using PASS. SAR QSAR Environ. Res..

[16] Dmitriev A., Filimonov D., Lagunin A., Karasev D., Pogodin P., Rudik A., Poroikov V. (2019). Prediction of Severity of Drug-Drug Interactions Caused by Enzyme Inhibition and Activation. Molecules.

[17] DrugBank. https://go.drugbank.com/.

[18] ADME Database. https://www.fujitsu.com/jp/group/kyushu/en/solutions/industry/lifescience/admedatabase/.

[19] Poroikov V.V., Filimonov D.A., Borodina Y.V., Lagunin A.A., Kos A. (2000). Robustness of Biological Activity Spectra Predicting by Computer Program PASS for Noncongeneric Sets of Chemical Compounds. J. Chem. Inf. Comput. Sci..

[20] DDI-Pred: Web-Service for Drug-Drug Interaction Prediction. http://way2drug.com/ddi/.

[21] Hansten P.D., Horn J.R. (2017). The Top 100 Drug Interactions A Guide to Patient Management.

